# ESR statement on new approaches to undergraduate teaching in Radiology

**DOI:** 10.1186/s13244-019-0804-9

**Published:** 2019-11-19

**Authors:** European Society of Radiology (ESR), Laura Oleaga, Marc Dewey, Roberto Iezzi, Franz Kainberger, Christiane M. Nyhsen, Carlo Catalano, Vlastimil Válek, Malgorzata Szczerbo-Trojanowska, Carmelo Messina, Fatih Seker

**Affiliations:** Vienna, Austria

**Keywords:** Radiology, Teaching, Undergraduate, Flipped classroom

## Abstract

Medical education is evolving and electronic learning (e-Learning) strategies have now become an essential asset in radiology education. Radiology education is a significant part of the undergraduate medical curriculum and the use of e-Learning in radiology teaching in medical schools is on the rise. If coupled with clinical decision support systems, e-Learning can be a practical way of teaching students clinical decision making, such as selecting the diagnostic imaging tests that are best suited in certain clinical scenarios.

The innovative concept of flipped classroom learning encourages students to work independently and maximises the application of learnt contents in interactive classroom sessions.

For integrated curricula with their student-centred, problem-based, and community-based design, an approach to systematically integrate radiology may be to define diagnostic reasoning as one of the core goals. Radiologists as teachers and scholars may understand themselves as experts in diagnostic reasoning and in mentoring how to make medical decisions.

Computer programs simulating the routine work are available and can be used to teach the recognition of anatomical structures and pathological patterns, and also to teach ultrasonography and interventional radiology, maximising patient safety.

## Key points

- Radiology teaching in medical schools is evolving; e-Learning modules must be integrated into the undergraduate teaching curriculum.
- Online independent preparation, prior to classroom sessions where learnt contents can be applied, is part of the new teaching approaches.
- Learning and teaching the appropriateness of imaging means a paradigm shift towards a more holistic approach of undergraduate education in radiology.
- Simulation-based training represents a key feature for undergraduate’s education.

## Introduction

Over the past several decades, the practice of radiology has undergone substantial changes, primarily related to the advances in imaging technology, the rise of importance of specific parts of diagnostic or interventional radiology, the changes in the infrastructure of healthcare delivery, and the evolution of reimbursement systems.

Rapid technological innovation in radiology has led to continuous advances that have been introduced in clinical practice and have caused a great change in the specialty.

While some universities have already implicated new technology and new methods of learning, many still find it difficult to implement substantial changes to the curriculum delivery.

The range of radiology teaching at medical schools has remained essentially unchanged. At the same time, it is unthinkable to make a diagnosis without computed tomography, magnetic resonance, ultrasound, and other imaging methods in the present day. What once were auscultation, palpation, percussion, and careful history taking are now computed tomography (CT), magnetic resonance (MR), ultrasound, and a properly completed order form for imaging studies.

Reorganisation of radiology education means drawing the students into radiology departments, infiltrating other courses with radiology lessons, and expanding the number of teaching hours. Students must learn many things in radiology, some of which are not directly related to the actual subject.

The importance of imaging methods in clinical practice must be reflected in the lectures. The teaching of radiology should be placed late in the study period, when students are equipped with sufficient knowledge of other clinical disciplines to understand the position of each imaging technique, the proper “cascade” of imaging techniques selection. It is worth to teach students that the old paradigm of choosing the less expensive and easy obtainable imaging method in the first line should not be the proper indication.

Radiology training programs around the world face a challenging task in both teaching a common knowledge base across all the imaging modalities and in imparting deep knowledge within each imaging domain. Today, medical school teaching of radiology includes anatomy, imaging, interventions, and new technology. Cross-sectional and virtual anatomy are extremely important.

Clinical procedures performed without such relevant anatomical knowledge could result in serious harm to patients. On the other hand, the contacts with radiology have to commence already during the anatomy lectures – after all, the foundation of any image evaluation is knowledge of anatomy, in fact, a very detailed anatomy.

Important parts of the training programs are radiological e-Learning activities [1–3]. E-Learning has been used recently in dental and general medicine curricula to support traditional learning methods. Effectiveness of e-Learning in radiology education when compared with traditional classroom learning methods is higher and students had positive attitude when using e-Learning, flipped classroom, problems solving scenarios, and advanced simulations.

## E-Learning

Education is evolving and the digital transformation has provided us with new tools for teaching [4] with and without image interaction possibilities [5]. New technologies and innovative approaches may help teaching radiology knowledge and skills to medical students [5]. Notably, e-Learning is also helpful in increasing skills and knowledge of radiographers about magnetic resonance imaging optimisation and artefact reduction [6]. Also, e-Learning solutions for radiological anatomy have been shown to help first-year medical students to review anatomy [7]. Moreover, so-called adaptive tutorials, which are online intelligent tutoring systems that provide a personalised learning experience, have shown in a cross-over study to significantly improve the understanding of diagnostic imaging in senior students [8]. Teaching radiology students by residents was facilitated in another study using web-based educational materials compared with traditional materials [9].

E-Learning activities are designed to individualise the learning experience [2, 3], in addition to improve the structure of knowledge and skills defined in the curriculum. This increased ability using e-Learning goes hand-in-hand with an increased clinical demand for better training students in selecting the most appropriate imaging test for the right patients at the right point in time (iGUIDE, etc.). Providing greater value to patient care may result from more consistent and evidence-based selection of imaging tests that are best suited for individual clinical scenarios [10].

Interactive learning methods are rarely used in most medical schools where didactic lectures are still widely used at present. It is, therefore, difficult to assess the learning outcomes associated with the new e-Learning approaches and to separate improvements from the general trend towards more interactive teaching. In a randomised analysis conducted at Charité in Berlin (Germany), it was found that guiding medical students towards imaging test selection resulted in significantly more appropriate tests being selected for the individual case scenarios [11]. Nevertheless, for a unique learning experience, it appears essential to have personal interactions with clinicians in a real-world hospital and outpatient care and to include a certain amount of humour in teaching radiology.

It is still important to address the role of e-Learning in radiology education and how to assess possibly improved outcomes with the use of new technologies and innovative approaches to teaching.

## Flipped classroom

Undergraduate radiology teaching classically involved didactic lectures, often to large groups of students, conveying volumes of entirely new information. Large group lectures do not facilitate the development of problem-solving skills required in radiology. Students in this scenario are passive recipients whilst the teacher is the “sage on the stage”, who is obliged to teach a medium grade of difficulty to the entire cohort of learners, often repeating the same PowerPoint lecture for years. When questioned, students greatly value interactive elements of teaching and small group sessions [12]. They are eager to participate and solve questions, preferably using real patient scenarios that are close to daily medical practice. This is not only perceived as the most interesting teaching method, but it also prepares students more effectively for the challenges of clinical decision-making they might encounter in their new role as junior doctors and, additionally, improves their ability to provide excellent patient care [13].

The flipped classroom approach encourages students to work independently to learn basic facts and concepts outside the classroom through various methods: watching recorded lectures, reading, and completing online education modules [14]. Students take on an active role in their learning pathway and this seems to achieve better results [9, 15, 16].

This type of methodology is perceived positively by students. The possibility of having unrestricted home access to pre-recorded video lectures and other materials enables students to learn at their own pace, pause and repeat as required, and go over the materials as many times as they wish. Difficult topics can be repeated, and additional resources can be used, if needed. This is a great advantage. The teacher, on the other hand, does not need to present the same lecture over and over again, they can record it once or find other suitable online materials (which would, of course, be carefully chosen and aimed at the level of the respective student groups). Instead of classic lectures, teachers can prepare interactive small group sessions, give students real clinical scenarios to solve, and become a “guide on the side”, helping students whenever needed at the precise individual level. Interactions in small groups with instant feedback during in-class sessions makes the flipped classroom a preferred education model. Different publications have shown that the flipped classroom approach can increase student-learning performance in comparison to traditional methods [17].

The flipped learning approach has a lot of potential, both for teachers and students, and will undoubtedly be developed further by most universities in the future. However, student motivation is essential. Without adequate home preparation, any classroom session based on previously acquired knowledge is bound to fail (and receive negative feedback). Therefore, excellent organisational skills are very important when introducing flipped learning. Precise timetables are recommended, detailing which sessions need to be prepared when, with several student reminders. Should this be deemed necessary, an entry test at the beginning of the lecture can be considered. Teachers need to prepare face-to-face sessions and have excellent communication skills, so that students can see the benefit of home preparation and really enjoy them [18]. In case students are not familiar with flipped teaching, it might be helpful to start with only some flipped elements.

## Problem solving scenarios

Teaching involves imparting medical knowledge and assisting trainees in developing competency and skills in patient care, helping them to drive themselves, make their own decisions, and develop personal relationships to achieve career goals. Medical schools have to integrate problem- and team-based learning into their curricula. One important aspect to be included in undergraduate education is the appropriate use of imaging studies; this should be incorporated as a subject in the undergraduate’s curriculum for medical schools [13, 14, 19].

Guidelines and appropriateness criteria for guiding imaging decisions can be used to provide high-value care.

Medical students will become the future leaders in health care and mastering these skills is critical for their future.

## Appropriate use of imaging: simulating diagnostic reasoning

Radiology, similar to its role in clinical medicine, should be the “backbone” of teaching and learning in medical curricula. For integrated curricula with their student-centred, problem-oriented, and community-based design, an approach to the systematic integration of radiology can be to define diagnostic thinking as one of the leading objectives and goals. In this sense, radiologists as teachers and scholars may understand themselves as experts in diagnostic reasoning and in mentoring how to make medical decisions. Such radiology core curriculum can be embedded in the integrated undergraduate curricula as one of the central disciplines and with high visibility to students and stakeholders.

The didactic technique of learning the skill of diagnostic reasoning is simulation, for which case-based scenarios can be developed with the aim of analysing clinical problems and establishing an appropriate hypothesis for appropriate referrals for imaging. Such case-vignettes may be regarded as virtual patients for active learning with different levels of complexity for beginners and advanced students. The focus may be set on imaging anatomy, radiation protection, pathology, patient safety and communication, or health economy. The design may be simple multiple-choice-questions, short-answer questions, more advanced forms with multimedia or augmented reality, or embedded in a clerkship. With virtual and augmented reality, immersing the learner in a virtual world is associated with an even higher level of active learner participation [10].

A comprehensive and successful approach for realising such simulation techniques for training diagnostic reasoning is the use of a didactic referral sheet in the form of a series of short answer questions which can be embedded in commonly used e-Learning platforms [20]. It has been shown that, with such case-based simulations, fifth-year students improve their diagnostic skills significantly [21].

Feedback can be given with high clinical relevance by using a computer assisted diagnosis system (ACR appropriateness criteria, ESRiGuide, or similar solutions) [21–23]. In this form, many aspects of reasoning, also referred to as interpretative skills and professionalism, can be trained. They encompass radiation protection, learning to estimate the pre-test probability, alerting in case of emergencies, establishing a clinical hypothesis, applying the rules of personalised medicine, patient safety and empowerment, and reflecting scenarios with view on health economics. Such toolkits embedded in e-Learning platforms may be used for self-assessment, in onsite seminars, or for written or oral examinations. This way, radiology teaching and learning can be realised as a course about appropriateness guidelines with a holistic approach to radiation protection [24].

Learning and teaching the appropriateness of imaging means a paradigm shift towards a more holistic approach of undergraduate education. The main challenge in this new educational culture is that students and radiologists have to include the topics of non-interpretative skills and professionalism. In addition to the traditional way of teaching image interpretation, consideration should be given to any potential consequences which may stem from personnel fault or resource malfunction [23].

## Simulator programmes

Simulator programmes have been proved to be effective in other field of interests, like aviation, nuclear power industry, and other medical specialties, like surgery, endoscopy, gynaecology, and emergency medicine. Based on these previous experiences, simulation-training models were also applied to ultrasonography and interventional radiology to create opportunities for trainees and teams to learn skills through deliberate practice in safety, away from patients. Virtual reality (VR) represents a computer-generated reconstruction of anatomical environment with tactile interactions and it enables operators not only to learn on their own mistakes without compromising the patient’s safety, but also to enhance their knowledge and experience. This model allows to overcome disadvantages of standard apprenticeship model in which essential technical skills are usually acquired during deliberate practice in patients. Some disadvantages of the standard apprenticeship model are: cost, duration of cases (taking longer), need for an expert mentor, and lack of uniformity, because the model is limited to only learning clinical cases that their patients’ present. It is necessary to underline that the medical sector also has to ensure lifelong learning, training, and professional development of doctors, with a mandatory role for starting, improving, and maintaining professional skills, so that simulation could also be useful not only for beginners but also for seasoned experts as a method of maintenance of certifications and development of new skills [25–27].

There are currently four common types of simulator programmes that can be used: computer-based learning modules, phantom and/or part-task trainers, computer assisted mannequins, and VR. Augmented reality is also used to visually and physically insert the user in the virtual environment to simulate medical procedures and scenarios [27]. Computer programmes simulating the routine work of a radiologist are available and can be used to teach recognising anatomical structures and pathological patterns in a variety of different imaging techniques. The difficulty level can be adapted to the student’s level [28]. In detail, haptic technology can be used to create these physical interfaces, as force feedback or vibration stimuli. It can also be used for complex procedures or for managing common complications with the ability to recognise errors and mimic the resultant physiologic effects with a repeated practice until proficiency metric are met.

Increasingly, simulation-based training has been used in ultrasonography and interventional radiology to maximise patient safety and ensure adequate students training. However, despite its ability to revolutionise clinical skill training, simulation still remains only a hypothetical possibility, mainly due to cost and artificial conditions of practice [25–27]. This potential can be hindered by sparse and non-uniform evidence of educational validity of commercially available simulation technology. Face validity of simulators is easy to assess, and many commercially available simulations have been validated. The ultimate goal in simulation research is to prove predictive validity and transference. This will lead to widespread implementation. To reach this aim, simulators must first be incorporated into programs where extensive research on devices and simulation-based training can be performed in order to include it in standardised interventional radiology and ultrasonography curriculum, to create a new hybrid educational paradigm that bolsters the safety, efficacy, and uniformity of medical education [25].

Simulator programmes have been proved to be effective in other specialties as a method for teaching. There are different types of simulator programmes that can be used including mannequin based or computer-based simulation programmes. VR simulation training has been found to be effective for surgical procedures as it allows trainees to develop skills before patient contact. Endovascular simulation procedures programmes have been used in vascular surgery courses to improve medical student performance with respect to technical skill, patient safety, and global performance assessment.

There are also ultrasound simulators available, that replicate the functions of a real ultrasound machine, using life-sized mannequins simulating a real-life procedure [29].

This methodology could be useful and can be applied for ultrasound and interventional radiology teaching.

## Conclusions

Medical schools and university hospitals face new challenges and opportunities in radiological education. The development of new learning tools requires adapting access to education. Teachers are not merely a source of information, but a guide for medical students in effective search for substantial information. The high level of academic teaching with quality content is a major factor in convincing students to become a radiologist.

It is essential to acquaint them with state-of-the-art radiological innovations and advanced teaching techniques and simulations to get them fascinated with clinical imaging.

## Data Availability

All data generated or analysed during this study are included in this published article.
